# Two cases reports: Severe liver injury caused by 1,2,3-trichloropropane poisoning

**DOI:** 10.3389/fpubh.2023.1171071

**Published:** 2023-04-14

**Authors:** Chen Li, Jinhua Hu, Haibin Su

**Affiliations:** Department of Liver Disease, Senior Department of Hepatology, The Fifth Medical Center of PLA General Hospital, Beijing, China

**Keywords:** severe liver injury, poisoning, hepatotoxicity, histology, 1,2,3-trichloropropane

## Abstract

**Background:**

1,2,3-trichloropropane (TCP) poisoning can induce liver damage in humans and animals, but reports of severe liver injury and its histology are rare. We presented two cases of 1,2,3-TCP inhalation poisoning resulting in severe liver injury confirmed by exposure history, toxicology, biochemical index and pathology.

**Case description:**

Two young male presented acute poisoning process with mild to moderate early symptoms, and developed severe jaundice and coagulation dysfunction after exposure to 1,2,3-TCP. The total bilirubin (TBIL) in case 1 and case 2 reached the peak value of 635.8 μmol/L and 437.1 μmol/L on the 25th and 22nd days, respectively. Their liver enzymes and international normalized ratio increased rapidly to peak and fell back, and TBIL remained at a high level. 1,2,3-TCP was detected in their blood, and their liver histology indicated severe necrosis of hepatocytes, infiltration of massive inflammatory cells, and cholestasis. They all finally recovered after a long time of treatment.

**Conclusion:**

The two cases in this study demonstrate that 1,2,3-TCP inhalation poisoning without any protective measures can induce severe liver injury in humans.

## Introduction

1,2,3-trichloropropane (TCP) is a colorless flammable liquid, which is widely used in agricultural and industrial productions. It is usually used as an industrial solvent for manufacturing of paints and chemicals, and an intermediate of pesticide (1, 2). Experiments in rats and mice suggest that 1,2,3-TCP is mainly metabolized by the liver. 1,2,3-TCP poisoning can damage liver, heart, respiratory system, central nervous system, and kidney, and can cause malignant tumors (3–5). However, both the English and Chinese literature reports on human injuries induced by 1,2,3-TCP poisoning are extremely rare. Only two case reports without histology described severe hepatotoxicity in humans caused by 1,2,3-TCP ingestion poisoning in English (6, 7). Early use of blood purification may be effective for patients with large exposure dose or critical illness (7). We reported two cases of 1,2,3-TCP inhalation poisoning induced by severe liver injury and confirmed by exposure history, toxicology, biochemical index and pathology, one of whom was combined with acute kidney injury (AKI) and sepsis. The main biochemical indicators which including liver enzymes (lactate dehydrogenase method and malic dehydrogenase method), bilirubin (vanadate oxidation method), and bile acid (enzyme cycle method) were tested in our hospital by the AU5800 automatic biochemical analyzer produced by Beckman Kurt Company (United States).

## Case description

### Case 1

**Figure 1:**
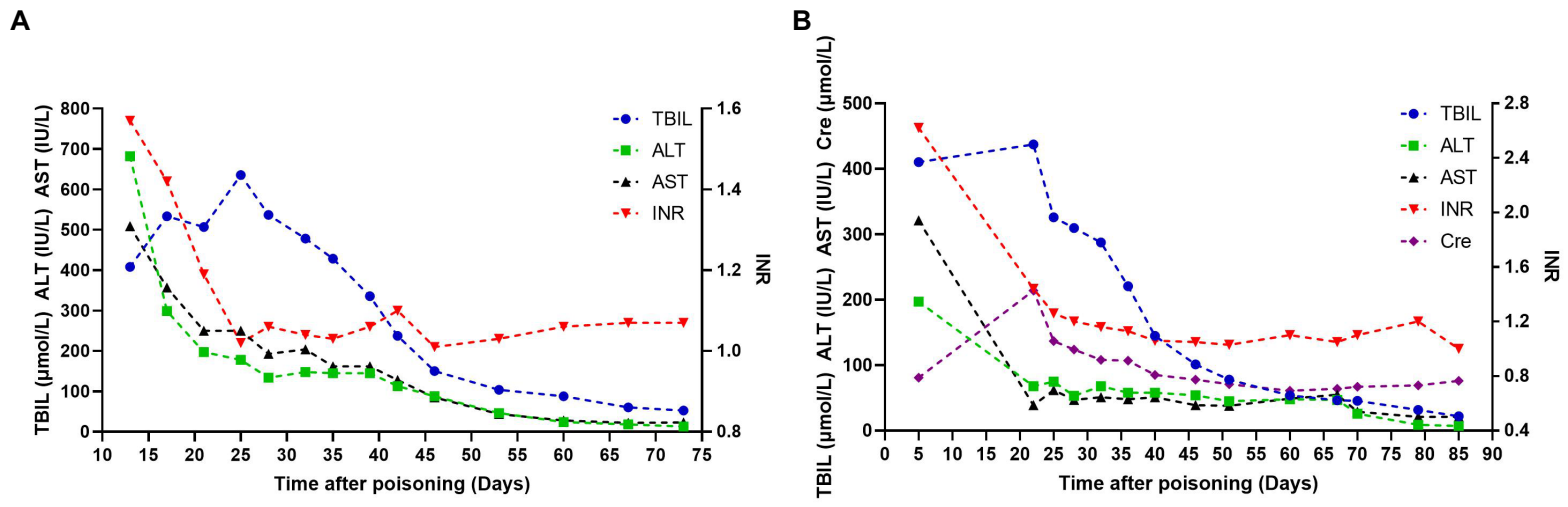
Dynamic trends of the main biochemical indexes of patients. **(A)** Represents case 1, **(B)** represents case 2.

**Figure 2:**
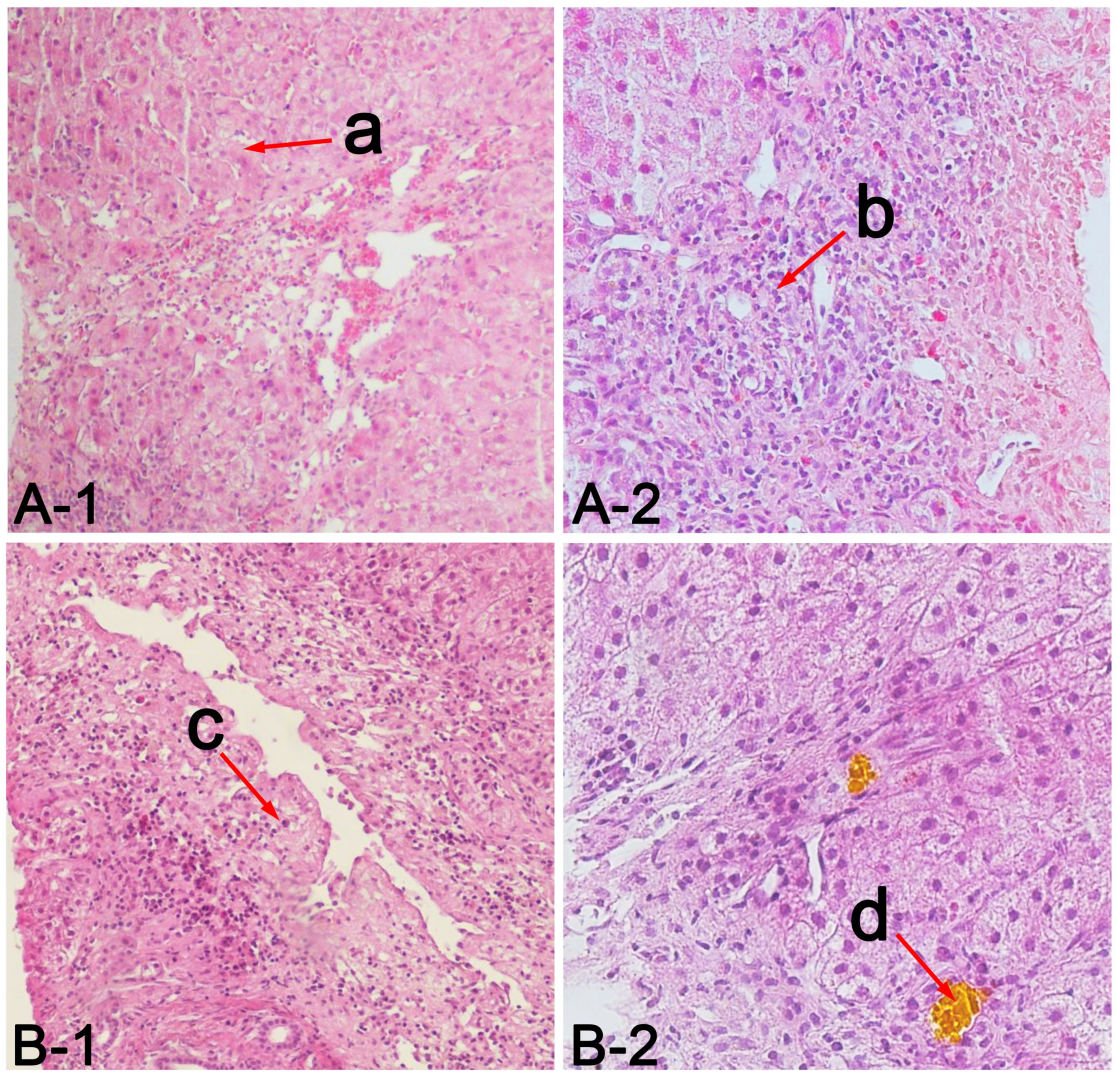
Liver pathological findings of patients. **(A)** represents case 1, **(B)** represents case 2, a in A-1 indicates diffuse hydropic degeneration, spotty necrosis and fusion necrosis of hepatocytes (hematoxylin and eosin, 100×), b in A-2 indicates numerous neutrophils, lymphocytes and eosinophils infiltration in the portal area (hematoxylin and eosin, 200×), c in B-1 indicates mesh stent collapse of hepatic lobule and submassive necrosis of hepatocytes (hematoxylin and eosin, 100×), d in B-2 indicates cholestasis in bile (hematoxylin and eosin, 200×).

A 20-year-old male college student majoring in chemical engineering from a city in northwest China was exposed to the nonventilated environment of industrial paint with pungent smell for 1 h without personal protective equipment (PPE). He had chest tightness and abdominal distension on the second day and developed jaundice and yellow urine on the fourth day. 1,2,3-TCP was positive in his blood with qualitative toxicological test by gas chromatography–mass spectroscopy (GC–MS) method (the column characteristics, country of origin, and model number were unknown), and other ingredients were not detected. On the 13th day, he developed nausea and vomiting, and the laboratory tests showed that the values of TBIL 408.3 μmol/L, direct bilirubin (DBIL) 295.8 μmol/L, alanine aminotransferase (ALT) 682 IU/L, aspartate aminotransferase (AST) 509 IU/L, and international normalized ratio (INR) 1.57. The patient was transferred to our hospital on the 17th day due to deterioration. The patient has no long-term history of alcohol and drugs intake and no family history of liver disease. The physical examination showed that his skin and sclera were severely yellow-stained. The laboratory tests showed the values of albumin (ALB) 45 g/L, TBIL 533.5 μmol/L, DBIL 411.8 μmol/L, ALT 299 IU/L, AST 357 IU/L, alkaline phosphatase (ALP) 168 IU/L, glutamyltransferase (GGT) 38 IU/L, total bile acid (TBA) 242 μmol/L, INR 1.42, creatinine (Cre) 84 μmol/L, white blood cell (WBC) 8.13 × 10^9^/L, hemoglobin (Hb) 155 g/L, and platelet (PLT) 232 × 10^9^/L. Hepatitis A, B, C, and E serology was negative. Cytomegalovirus and Epstein–Barr virus serology was negative. Autoimmune antibodies were negative, the level of ceruloplasmin and serum iron were normal. The patient received glutathione (2.4 g/day), magnesium isoglycyrrhizinate (150 mg/day) and hepatocyte growth-promoting factors (120 ug/day) to protect hepatocyte, vitamin K1 (10 mg/day) to improve coagulation function. His laboratory indicators of ALT, AST, and INR showed rapid downward trends during the treatment. His TBIL peaked at 635.8 μmol/L on the 25th day and dropped below 10× the upper limit of normal (ULN) on the 46th day (Figure 1A). On the 67th day, the patient received liver biopsy, which indicated diffuse hydropic degeneration, spotty necrosis and fusion necrosis of hepatocytes, numerous neutrophils, lymphocytes and eosinophils infiltration in the portal area, and cholestasis in bile (Figure 2A). He was discharged on the 73th day, and the laboratory tests showed the following values: TBIL 52.6 μmol/L, DBIL 39.1 μmol/L, ALT 13 IU/L, AST 23 IU/L, ALP 120 IU/L, GGT 24 IU/L, TBA 19 μmol/L, and INR 1.07. One year after discharge, the patient was in good health and normal life.

### Case 2

An 18-year-old male worker from a village in central China was exposed to the industrial paint environment with pungent smell for 8 h a day without PPE for 3 days, with intermittent chest tightness. On the 4th day, he developed jaundice, yellow urine, and poor appetite. On the 5th day, his laboratory tests showed the following values: ALB 28.9 g/L, TBIL 410.4 μmol/L, DBIL 292.8 μmol/L, ALT 197 IU/L, AST 321 IU/L, ALP 186.5 IU/L, GGT 53.6 IU/L, INR 2.62, Cre 81 μmol/L, WBC 3.2 × 10^9^/L, Hb 106 g/L, PLT 50 × 10^9^/L. 1,2,3-TCP was positive in his blood without other ingredients with qualitative toxicological test by GC–MS method (the column characteristics, country of origin, and model number were unknown), and other ingredients were not detected. The patient received 14 days of corticosteroid (40–60 mg/day) to treat severe jaundice in the local hospital. The patient was transferred to our hospital on the 22nd day due to the ineffectiveness of corticosteroid and deterioration. The patient has no long-term history of alcohol and drugs intake and no family history of liver disease. The physical examination showed that his skin and sclera were severely yellow-stained, his lower limbs had pitting edema. The laboratory tests showed the following values: ALB 25 g/L, TBIL 437.1 μmol/L, DBIL 295.3 μmol/L, ALT 68 IU/L, AST 39 IU/L, ALP 144 IU/L, GGT 37 IU/L, TBA 89 μmol/L, INR 1.44, blood urea nitrogen (Bun) 20.66 mmol/L, Cre 214 μmol/L, WBC 15.64 × 10^9^/L, Hb 101 g/L, PLT 79 × 10^9^/L. Hepatitis A, B, C, and E serology was negative. Cytomegalovirus and Epstein–Barr virus serology was negative. Autoimmune antibodies were negative, the level of ceruloplasmin and serum iron were normal. The patient had persistent fever during hospitalization, and the blood culture confirmed the presence of sepsis caused by *Staphylococcus epidermidis*. He received glutathione (2.4 g/day), magnesium isoglycyrrhizinate (150 mg/day) and hepatocyte growth-promoting factors (120 ug/day) to protect hepatocyte, vitamin K1 (10 mg/day) to improve coagulation function. He also received albumin (10–20 g/day), rehydration (normal saline and Ringer’s solution, 1000–2000 ml/day) to improve renal function, antibiotics (piperacillin sodium and tazobactam sodium, 13.5 g/day) to control sepsis after admission. His AKI and sepsis resolved, and laboratory indicators of ALT, AST, INR, and Cre gradually improved. The level of TBIL peaked at 437.1 μmol/L on the 22nd day and descended below 10 × ULN on the 40th day (Figure 1B). On the 68th day, the patient received liver biopsy, which indicated mesh stent collapse of hepatic lobule and submassive necrosis of hepatocytes, numerous neutrophils, lymphocytes and eosinophils infiltration in the portal area, and cholestasis in bile (Figure 2B). He was discharged on the 85th day after poisoning, and the laboratory tests showed the following values: ALB 34 g/L, TBIL 22.0 μmol/L, DBIL 17.4 μmol/L, ALT 7 IU/L, AST 21 IU/L, TBA 15 μmol/L, INR 1.0, Bun 3.8 mmol/L, Cre 76 μmol/L. One year after discharge, he maintained normal liver and kidney functions, and a normal quality of life.

## Discussion

As a persistent chemical environmental pollutant, 1,2,3-TCP can act on humans and animals through ingestion poisoning, inhalation poisoning, and percutaneous poisoning. However, most of the studies on the toxicity of 1,2,3-TCP are animal poisoning experiments. A few studies on humans without pathology show that 1,2,3-TCP poisoning can lead to hepatotoxicity (6–8), painful peripheral neurotoxicity (9), and acute leukoencephalopathy (10). Some patients with 1,2,3-TCP poisoning may not have undergone toxicological test, so missed diagnosis can occur in the real world. A study on 18 cases of transient 1,2,3-TCP inhalation poisoning showed that respiratory symptoms, gastrointestinal symptoms and painful peripheral neurotoxicity were the most common clinical manifestations, and only one patient had obvious liver injury (8). The previous two patients with acute severe liver injury were all caused by accidental 1,2,3-TCP ingestion poisoning, and they all had nausea, vomiting, and disturbance of consciousness within 1 h. The ALT, AST, and TBIL of the 45-year-old male rapidly increased to 2916.4 IU/L, 6501.8 IU/L, and 105.9 μmol/L, respectively, within 2 days after poisoning, accompanied by coagulation dysfunction, AKI, and shock. He continued to deteriorate after comprehensive treatment (6). The liver enzymes of another 56-year-old female reached their peaks (ALT 5100 IU/L and AST 5290 IU/L) on the 4th day and decreased rapidly on the 9th day. Her TBIL levels elevated gradually and peaked at 351.1 μmol/L on the 20th day. Her condition was improved after receiving hemoperfusion and plasma exchange (7).

Compared with the 1,2,3-TCP ingestion poisoning patients, the two patients we reported had some clinical characteristics. First, they had no basis for liver disease and suffered liver damage after exposed to 1,2,3-TCP without any protective measures. They had attracted the attention of clinicians due to occupational poison exposure. Toxicity tests confirmed that their blood contained 1,2,3-TCP. Viral hepatitis, alcoholic liver disease, drug-induced liver injury, autoimmune hepatitis, hemochromatosis and Wilson’s disease were excluded by biochemical index and pathology. These results suggest that there is an obvious causal relationship between severe liver injury and 1,2,3-TCP poisoning. Second, their early symptoms were mild to moderate without coma and shock. They presented acute poisoning process and showed signs of hepatotoxicity on the 4th day after poisoning. Their ALT, AST, and INR levels increased rapidly to peak and then decreased rapidly, but the peak values of ALT and AST were lower than those previously reported (6, 7). However, their TBIL remained at a high level, reaching a peak in the 22nd-25th days and falling back below the 10 × ULN in the 40th–46th days. We speculate that this phenomenon may be related to different poisoning modes and concentration of 1,2,3-TCP. Unfortunately, due to the limited conditions of the local hospital, the two patients did not complete the quantitative toxicological test in the early stage of poisoning to evaluate the concentration of 1,2,3-TCP and eliminate the false positive results. In addition, previous study showed that 1,2,3-TCP was a contaminant of concern in groundwater and drinking water (11), so it was also meaningful to detect 1,2,3-TCP in the water source of patients’ settlements to exclude the possibility of chronic poisoning. Third, liver is the most important organ for metabolism of 1,2,3-TCP, and the reactive intermediates formed in the metabolic process can cause DNA damage and dysfunction of hepatocytes by binding to protein in animal studies (2, 12). Further, histology in rats showed that 1,2,3-TCP poisoning can lead to the increase in liver weight and fatty liver, as well as the mild to moderate hepatic necrosis with increasing incidence as related to dose (4, 13, 14). Our human histological studies confirmed that 1,2,3-TCP poisoning resulted in severe necrosis of hepatocytes, infiltration of massive inflammatory cells, and cholestasis, which indicated that their liver has undergone a severe attack and need a long time to recover. Therefore, the biochemical indexes of these two patients recovered to meet the requirements of percutaneous liver biopsy until the 67th and 68th days after poisoning. Furthermore, their hospitalization period was more than 70 days. Fortunately, clinical follow-up showed that both patients eventually recovered without affecting their quality of life.

## Conclusion

In conclusion, we reported two cases of severe liver injury caused by occupational poisoning of 1,2,3-TCP, and their recovery time was very long. The severe hepatotoxicity and a series of complications caused by 1,2,3-TCP should be alerted. Exposure history, toxicology, biochemical index and pathology are very important for diagnosis. Glutathione can detoxify the reactive intermediates caused by 1,2,3-TCP poisoning (12) and should be used for first-line treatment. Appropriate PPE should be used for people exposed to 1,2,3-TCP.

## Data availability statement

The original contributions presented in the study are included in the article/supplementary material, further inquiries can be directed to the corresponding authors.

## Ethics statement

The studies involving human participants were reviewed and approved by the Fifth Medical Center of PLA General Hospital. The patients/participants provided their written informed consent to participate in this study.

## Author contributions

CL, JH, and HS participated in the treatment of the two patients, designed the study, and collected the data. CL wrote the first draft with assistance from JH and HS. JH and HS edited the final manuscript. All authors contributed to the article and approved the submitted version.

## Funding

This work was supported by National Key R&D Program of China (2021YFC2301801).

## Conflict of interest

The authors declare that the research was conducted in the absence of any commercial or financial relationships that could be construed as a potential conflict of interest.

## Publisher’s note

All claims expressed in this article are solely those of the authors and do not necessarily represent those of their affiliated organizations, or those of the publisher, the editors and the reviewers. Any product that may be evaluated in this article, or claim that may be made by its manufacturer, is not guaranteed or endorsed by the publisher.
